# Connecting Macroscopic Observables and Microscopic Assembly Events in Amyloid Formation Using Coarse Grained Simulations

**DOI:** 10.1371/journal.pcbi.1002692

**Published:** 2012-10-11

**Authors:** Noah S. Bieler, Tuomas P. J. Knowles, Daan Frenkel, Robert Vácha

**Affiliations:** 1 Laboratory for Physical Chemistry, ETH Zürich, Zurich, Switzerland; 2 Department of Chemistry, University of Cambridge, Cambridge, United Kingdom; 3 National Centre for Biomolecular Research, Faculty of Science and CEITEC - Central European Institute of Technology, Masaryk University, Brno-Bohunice, Czech Republic; University of Illinois, United States of America

## Abstract

The pre-fibrillar stages of amyloid formation have been implicated in cellular toxicity, but have proved to be challenging to study directly in experiments and simulations. Rational strategies to suppress the formation of toxic amyloid oligomers require a better understanding of the mechanisms by which they are generated. We report Dynamical Monte Carlo simulations that allow us to study the early stages of amyloid formation. We use a generic, coarse-grained model of an amyloidogenic peptide that has two internal states: the first one representing the soluble random coil structure and the second one the 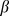-sheet conformation. We find that this system exhibits a propensity towards fibrillar self-assembly following the formation of a critical nucleus. Our calculations establish connections between the early nucleation events and the kinetic information available in the later stages of the aggregation process that are commonly probed in experiments. We analyze the kinetic behaviour in our simulations within the framework of the theory of classical nucleated polymerisation, and are able to connect the structural events at the early stages in amyloid growth with the resulting macroscopic observables such as the effective nucleus size. Furthermore, the free-energy landscapes that emerge from these simulations allow us to identify pertinent properties of the monomeric state that could be targeted to suppress oligomer formation.

## Author Summary

A number of normally soluble proteins can form amyloid structures in a process associated with neurodegenerative diseases such as Alzheimer's and Parkinson's diseases. Mature amyloid structures consist of large fibrils containing thousands of individual proteins aggregated into linear nanostructures; there is increasing evidence, however, that the toxic species responsible for neurodegeneration are not the mature fibrils themselves but rather lower molecular weight precursors commonly known as amyloid oligomers. Unfortunately, these early oligomers are commonly thermodynamically unstable and of nanometer scale dimensions, factors which make them highly challenging to probe in detail in experiments. We have used computer simulations of a model inspired by Alzheimer's Abeta peptide to investigate the early stages of protein aggregation. The results that we obtain were shown to fit Oosawa's polymerization theory, a finding which allows us to provide a connection between the microscopic molecular parameters and macroscopic growth. One crucial parameter is size of the nucleus, i.e. the basic oligomer existing at origin of the formation of each fiber. We have revealed a path for the formation of this nucleus and validate its size by several methods. Our results provide fundamental information for influencing the early stages of amyloid formation in a rational manner.

## Introduction

A wide range of normally soluble proteins and peptides are known to have a propensity to aggregate into 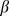-sheet rich amyloid fibrils. Such structures do sometimes posses functional roles, for instance as functional coatings and catalytic scaffolds [1]. However, more often than not, the formation of amyloid structures is a pathogenic event – it is the hallmark of a range of neurodegenerative disorders, including Alzheimer's and Parkinson's diseases [2], [3]. A diversity of experimental [4]–[9], and theoretical [10]–[15] approaches have been developed to probe the mechanisms of amyloid formation. Such studies have shed light on the structural and kinetic aspects of amyloid growth; it has, however, proved to be very challenging to characterise the very early stages of this reaction, in particular the primary nucleation events and the subsequent formation of low relative molecular weight oligomers. Yet there is substantial evidence that these small oligomers, rather than mature fibrils act as neurotoxins, and are implicated in the pathological cascades that underlie neurodegeneration [16]–[18]. Hence, despite the technical challenges associated with the study of this phenomenon [19], [20], a quantitative understanding of the kinetics of oligomer formation is of great practical and fundamental importance in the context of neurodegeneration and more generally in relation to aberrant protein self-assembly.

The growth of linear aggregates following a nucleation event is described in its simplest form by the classical theory of nucleated polymerisation, developed by Oosawa originally to study the formation of cytoskeletal filaments [21]. Within this framework, the time dependence of the mass fraction 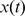 of the fibrils can be expressed as follows [21], [22]:
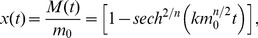
(1)where 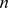 is the nucleus size, 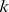 is an effective rate constant which contains contributions 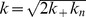 from both the nucleation rate 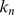 and the elongation rate 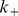 of the filaments, 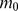 is the initial mass concentration of the monomers, 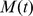 the mass concentration of the fibrils and 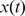 the mass fraction of the fibrils. The key parameters in Oosawa's theory are 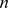 and 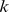, since 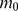 is known a priori. This formalism has been extended by Ferrone, Eaton and coworkers in their pioneering studies of the aggregation of sickle haemoglobin to include secondary nucleation events such as filament fragmentation and surface catalysed nucleation [23], [24]; in the present paper we focus on homogeneous nucleation which is crucial in the formation of the primary nuclei.

Several groups have reported numerical simulations of the nucleation and growth of amyloid fibrils [25]–[35]. Such simulations provide invaluable microscopic insight into the mechanism of amyloid formation. The focus of the present paper is different: we wish to compute quantities that are directly accessible to experiment, such as the time dependence of fibril formation, and apply to these quantities the same analysis that is applied to experimental data. This approach allows us to test whether a reliable estimate of the critical nucleus size can be obtained by fitting the experimental fibril-growth curve to an analytical approximation, and sheds light on the microscopic and mechanistic interpretation of the nucleus size. In addition, our simulations allow us to follow in detail the pathway by which amyloid fibril nucleation takes place and shed light on the condensation-reorganisation mechanism that underlies the primary nucleation process [36]. Since primary nucleation processes are very rare events, and the critical nucleus is by definition the species after the highest free energy barrier and therefore lowest relative concentration within the aggregation pathway, simulations are currently the most fruitful avenue to access the structural determinants of the critical nucleus and to follow its formation and the subsequent conversion to amyloid fibrils.

In the present paper we study fibril nucleation by considering a highly simplified model of amyloidogenic peptides that captures the salient features of this self-assembly process. A key property of amyloidogenic peptides is that they change conformation when converting from their normal soluble form into the amyloid state [3]. Our model has two internal states: one (denoted the 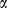-state) has weak intra-peptide interactions and represents the soluble random coil structure, the other (the 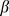-state) has a significantly higher intrinsic internal energy, but has stronger interpeptide attractions. The higher internal energy of the 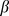-state results from the loss of conformational degrees of freedom relative to the 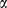-state, and its higher propensity to form inter-peptide contacts is a consequence of the availability of the residues for hydrogen bonding with neighboring peptides in the 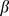 sheet conformation. In this picture, fibril nucleation takes place once the free-energy gain due to aggregation compensates the free energy cost of converting the monomers to the 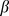-state. The model that we use is based on a peptide model reported in ref [37]. that has been extended to account for the two-state nature of amyloidogenic peptides (see section methods). Because this model is simple and therefore computationally highly tractable, it allows us to study the behavior of large numbers of peptides. Specifically, we can use it to compute the fibrillar growth profile and the free-energy landscape for oligomer formation. We used dynamic Monte Carlo simulations to generate trajectories for an ensemble of peptides in a system with periodic boundary conditions. In what follows, we relate the properties of the present model system to solutions of A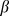 peptides with a length of 40–42 amino acid residues, which are the major components of the aberrant deposits found in connection with Alzheimer's disease [2], [3]. With this mapping, the conditions of our simulations correspond to a concentration range of 0.2–8 mM. For more details on the model and the simulation method, see section methods.

## Methods

We used Dynamic Monte Carlo (DMC) [38]–[41] for our simulations. In this method, the displacements, that can occur are so small, that no unphysical moves can occur. Moreover, the parameters of individual moves (translation and rotation) are fitted to experimental timescales such as diffusion and rotational constants. The values were taken from one of the most studied amyloid forming peptides A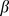 and were averaged between its 40 and 42 amino acid long forms (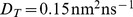 and 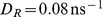 at 300 K) [42]. Hence, maximum displacement was d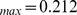 nm and maximum rotation 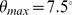 and consequently the time of our simulation step 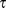 (when on average all particles move, i.e., sweep) can be roughly related to 0.02 ns.

**Figure 1:**
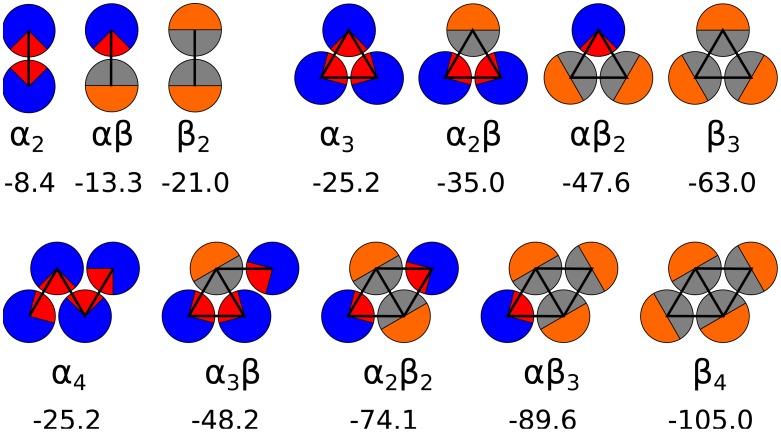
The configurations of small oligomers in the interaction minima with marked interactions and the total enthalpic contributions. The subscript and the size of the patch denotes the internal state of the PSC mode (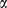: random coil and 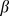: 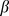-sheet).

The amyloidogenic peptides were modeled as Patchy Spherocylinders (PSC) [37], i.e. cylinders with hemispherical caps at both ends and with an attractive stripe on its side. As was shown in ref. [37] such particles can either occur in an oligomeric form or assemble into amyloid-like structures with two filaments, depending on the model parameters. Unlike the model described in ref. [37], the present model peptides can occur in two possible states: the first one (denoted as the 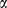-state) corresponds to the random coil conformation of peptide in solution [43]; the second state (called 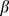-state) corresponds to the 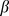-sheet structure found in the fibrils. The free energy difference corresponding to change from the 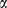 to the 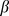-state, is denoted by 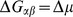. In what follows, we chose 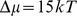. These values were chosen to reflect the fact that, in experiments, amyloidogenic proteins are typically not found at detectable concentrations in the 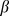-sheet conformation in solution [44], [45]. The attractive stripe is responsible for self-assembly and the cross-section of small oligomers in ideal conformation are depicted in Figure 1. Chirality was introduced into the model in order to reproduce the relatively long persistence length of the amyloid fibrils. This was achieved by rotating the attractive patch 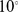 off the cylinder axis around the vector connecting the middle of the cylinder axis with the middle of the patch.The attractive stripe was thus misaligned with the body of the spherocylinder represented by repulsive potential.

The aspect ratio of the PSC was chosen to mimic the elementary 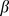-sheet unit of A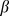 peptides with dimensions 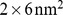. We use an implicit-solvent model where the interaction between the attractive stripes (patches) on different peptides effectively includes all possible interactions such as hydrophobic interaction, hydrogen bonds, salt-bridges, etc. The potential minimum of two interacting 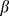-states was −21 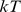 with an attractive stripe size of 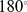 running length-wise in order to mimic the interaction potential of A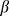 peptides [11] and being able to form cross beta fibrils. We considered both a chiral and a non-chiral version of the model.

The 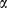-states were interacting with a minimum of −8.4 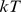 and had a patch size of 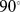, which was inspired by the hydrophobic patch of the random coil structure covering 25% of its surface [46] and its presence mainly in monomeric form in solution. The interaction between 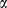 and 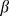 state was calculated using Berthelot's rule [47]. The interaction between the particles is effective, i.e. taking into account all the interactions.

The probability of a PSC to attempt to switch its conformation from the 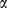-state to the 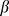-state or *vice versa* was 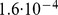 per particle move. The value was estimated based on the rearrangement time of a polypeptide with a size of 18 Kuhn lengths [5]. The fibrillar growth with switching probabilities one order of magnitude larger or smaller was without any significant difference.

In all our simulations we employed an NVT ensemble and periodic boundary conditions. The systems contained 600 PSC with the box sizes (in nm) of 50, 75, 100, 125, 150 and 175 corresponding to concentrations (in mM) of 7.97, 2.36, 1.00, 0.51, 0.30, and 0.19. For each concentration at least three separate runs were conducted with different random initial configurations and the obtained growth profiles were averaged over all runs. The size of a fibril for the relative mass profiles was defined as all oligomers with a size of at least four monomers.

## Results

### Fibrillar growth

**Figure 2:**
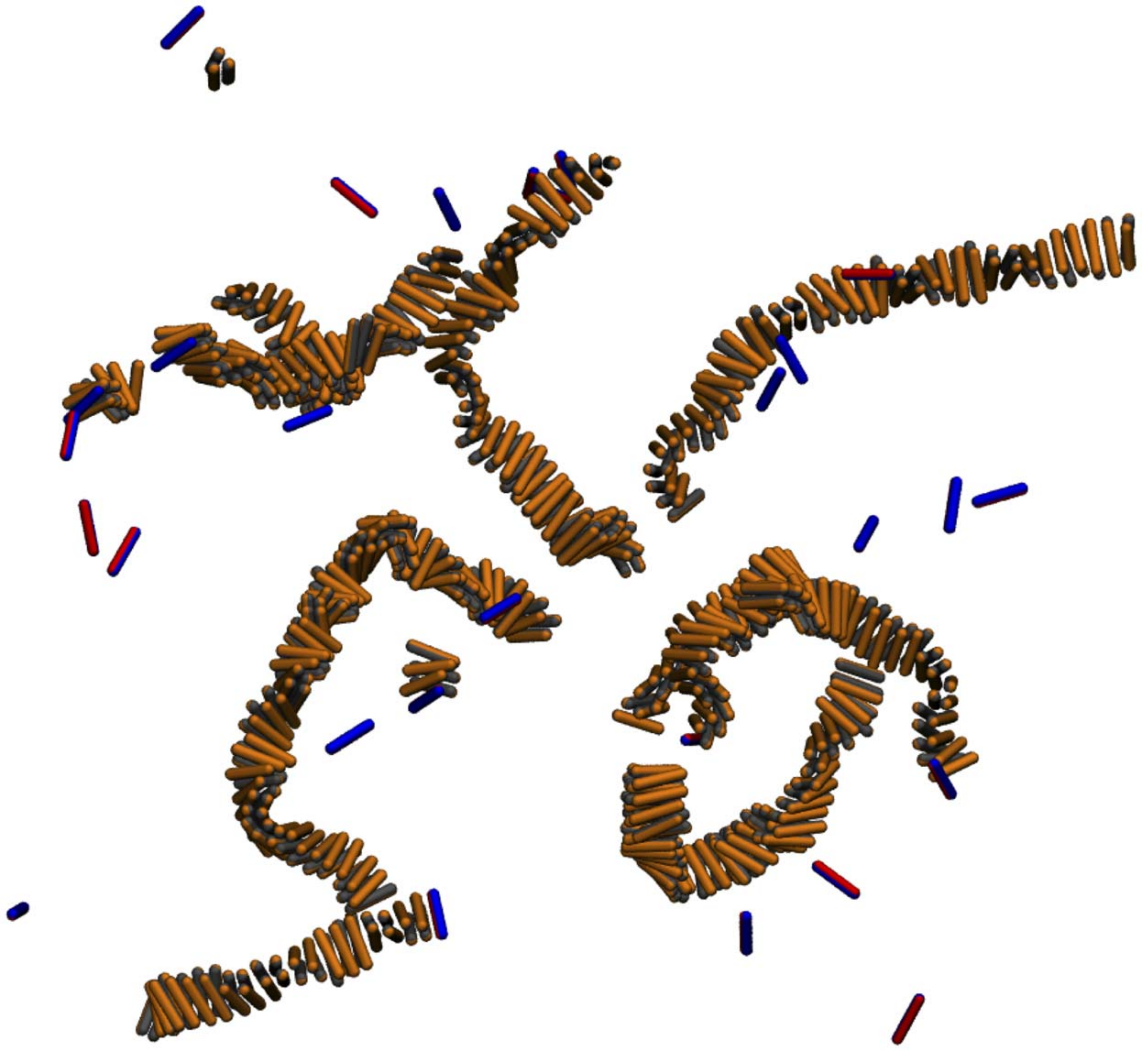
Representative snapshot of the simulation box in the later stage of the fibril growth. The blue/red particles are in the random coil state, while the orange/grey particles are in the 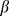-sheet state. Note that the particles in the random coil state are mostly monomers in solution or at the end of the fibres, while the 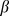-sheet are forming chiral cross stacked fibrils.

**Figure 3:**
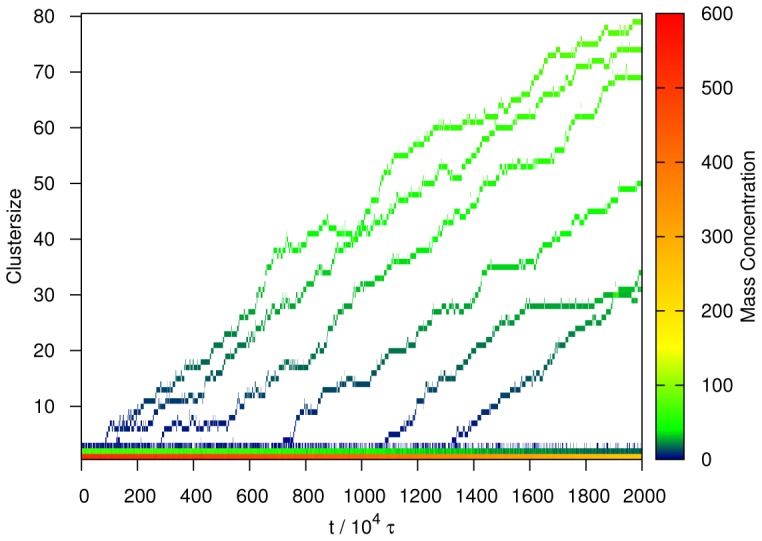
The time dependent distribution of oligomer mass, which captures the growth of individual fibers during one simulation with 0.51 mM peptide concentration. At the bottom, one can see the decreasing concentration of monomers (red/yellow), dimers (green/blue) and trimers (blue). Importantly, tetramers developed into a full fiber in every case. Only the beginning of the simulation is shown for clarity.

A representative snapshot of the late stage of a simulation of fibril growth is shown in Figure 2: it reveals that aggregation results in fibrillar species with a morphology similar to that observed in experiments [48]. Our simulations allow us to follow the time dependence of the aggregation number and hence of the mass of individual aggregates as they grow. Figure 3 shows a representative time trace. Initially the system is in a purely monomeric state. As a result of the collision of two peptides, a dimer can be formed. The dimer can either dissociate into monomers, or can grow to a trimer through monomer addition. The oligomers with an aggregation number below four are highly dynamic and interconvert readily between different aggregation states, including dissociation to monomer. However, tetramers, once formed, always develop into a fiber, which suggests that the size of the critical nucleus is 4 or just below. In what follows, every oligomer containing four or more monomers is counted as a fiber. At later times, as larger aggregates emerge, the numbers of monomers and oligomers (dimers and trimers) decreases through the incorporation of peptides into larger structures.

**Figure 4:**
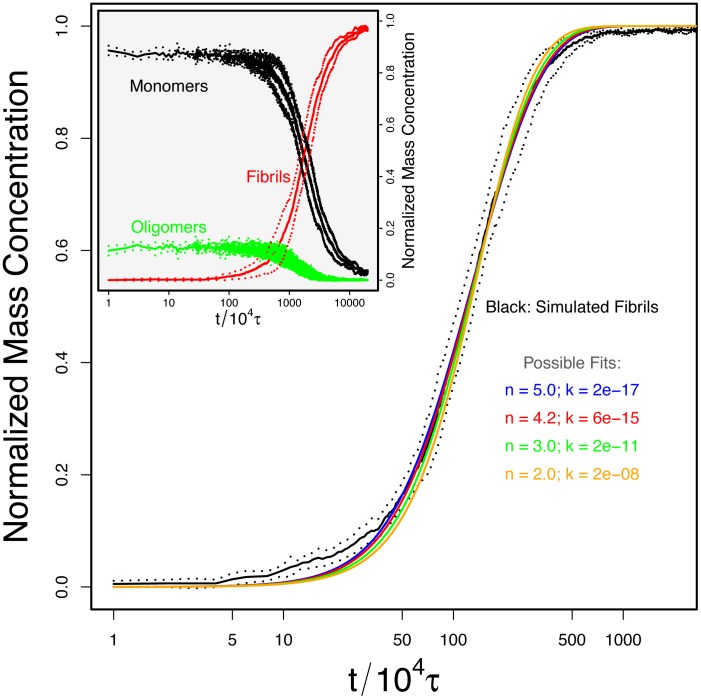
The fibrillar growth from our simulation fitted by Oosawa's theory with varying nucleation size. All the theoretical curves displayed with non-black colors were able to fit the simulation data very well by varying the growth rate from 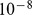 to 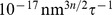 and the nucleus size from 2.0 to 5.0; this overfitting can only be resolved by performing global fits as shown in Fig. 5. The inset shows the time evolution of the mass concentrations of monomers (black), oligomers (dimers and trimers, green) and fibrils (aggregates with an aggregation number greater than four, red) for a simulation with a concentration 0.51 mM. The dots display the standard deviation calculated from the averaging of the five runs with different random initial configurations.

**Figure 5:**
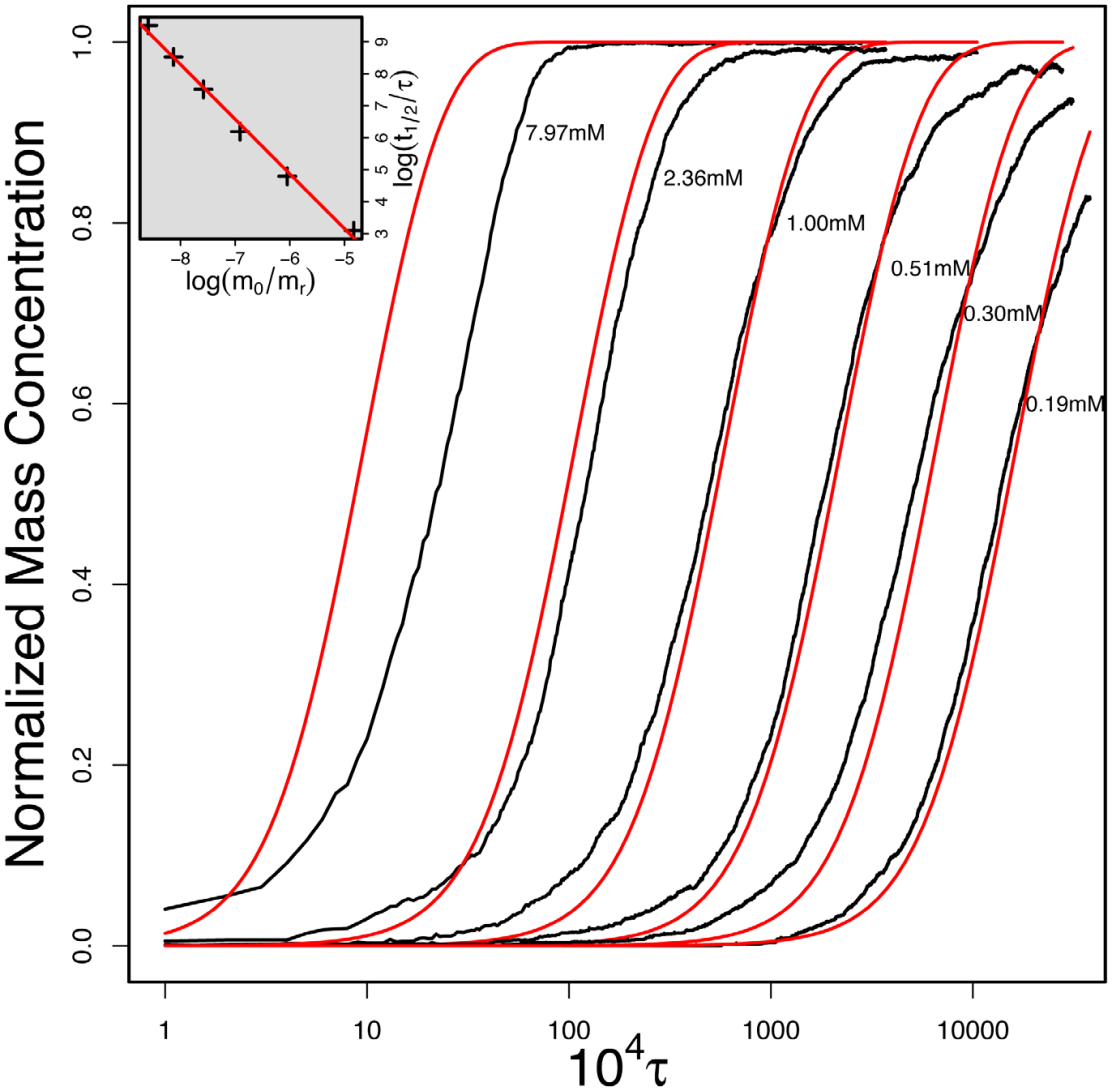
The fibril growth from our simulation and corresponding fit by Oosawa's theory. The peptide concentrations are from left to right 7.97, 2.36, 1.00, 0.51, 0.30, and 0.19 mM. The fitted size of nucleus and growth rate are 3.8 particle and 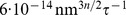 respectively. The inset shows the fit to obtain the nucleus size 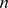 via the halftimes according to Eq. 2. The logarithmic time scale tends to visually over-emphasize the differences between the global fit (red curve) and data (black curve) at short time (highest concentrations).

Figure 4 shows the average increase in the aggregate mass concentration obtained at a fixed concentration (0.51 mM). We first tested whether these data could be fitted to the Oosawa theory. We find that, as is the case for experimental studies [15], analysis of the system under a single set of conditions does not yield strong enough constraints to allow a reliable estimate of the parameters in Oosawa's theory to be obtained. Indeed, fits of similar quality were obtained with critical nucleus sizes varying between 2.0 and 5.0. This finding therefore highlights the difficulty in resolving microscopic parameters, in this case the critical nucleus size, from macroscopic bulk data at a single concentration since a similar shape of the curve can be obtained for different combinations of the nucleus size and the nucleation rate. These two parameters can be disentangled, however, when data at different concentrations are considered. The average growth profiles of the simulation with different initial monomer concentrations were simultaneously fitted to Eq. 1 (see Figure 5). The best fit was obtained for a critical nucleus size 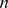 = 3.8 and a growth rate of 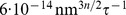, where 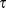 is the unit time of our simulation roughly corresponding to 0.02 ns (see Methods). We note that, in this formulation, 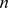 corresponds to the first species in the aggregation pathway that has a higher than 50% probability to grow into a fibril.

The results of the simulation also allow us to test the connections between the characteristic scaling behaviour [15], [22], [23], [49] of the half-time 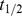 and the critical nucleus size that follows from Eq. 1 given as the powerlaw:
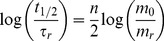
(2)where 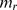 is the standard concentration and 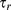 is the half-time of an aggregation reaction at this concentration. Following this procedure we obtain a slightly smaller estimate for the critical nucleus size: 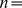
3.4.

**Figure 6:**
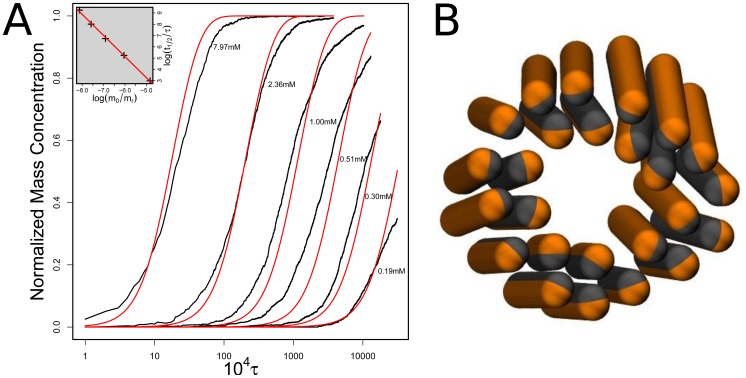
The rigidity of the fibers can be reduced by removing the chirality of the employed model. In such systems, the fibers can bend to form a ring (depicted as a simulation snapshot in part B), which does not have a loose end available for further growth. This effects the whole growth process (shown in part A as a relative fiber mass concentration depending on time) and it results in a deviation from a global fit to Oosawa's theory, especially at later stages of the fibrillar growth, since there is no such effect included in theory. The fitted values are 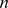 = 4.0 and 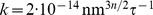. The black curve represents simulation data and the red curve is the fit.

To test the sensitivity of Oosawa's theory in identifying processes other than nucleation and growth, we also employed a model characterized by the absence of chirality compared to the model used previously (see Methods). In such a system, the persistence length of the polymers is low for bending mode parallel to the narrow dimension of the rectangular cross-section. In Figure 6A we show that there is a systematic deviation from the fit according to Oosawa's theory. In particular, the growth is always faster at the beginning and slower at the end of the growth than the fit. The reason is depicted in Figure 6B, where we can see that a flexible fiber can bent into a ring like structure, thereby effectively removing a potential growing site from the system (i.e., the fibrillar end is not accessible for further monomer addition). The fusion of fibrils, which we observed for both the non-chiral and the chiral models, also leads to a decrease in the late-stage rate of growth of fibrils. Neither ring formation nor fusion are accounted for in Oosawa's theory, and this fact could explain the slight deviation of the simulation data from the fit shown in Figure 6. Note that at lower concentrations not all the monomers are depleted from the bulk at the end of simulation. This is also the case in the chiral model and reflects the finite probability for monomers to dissociate from the aggregates leading to an equilibrium between the monomeric and aggregated forms of the peptides as is observed in experiments [50].

### Free energy landscape of oligomers

The simulations allow us to follow the time evolution of single aggregates as they form. This information makes it possible to relate the macroscopic average quantities such as the scaling exponents characterising the lag-time, to microscopic events taking place on the level of individual peptides. Based on classical nucleation theory, the free energy profile along the reaction coordinate (aggregation number) increases from the monomer up to a maximum, after which it monotonically decreases under supersaturated conditions. The point of the maximum free energy is related to the size of the critical nucleus. We define the nucleus as the first oligomer after this free energy barrier, in other words the nucleus is the smallest oligomer, which has a higher probability to grow than to shrink.

In order to look at the nucleation from yet another perspective, we constructed a free energy landscape for the different kinds of oligomers up to tetramer using the following procedure: The free energy of a mixed oligomer 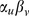 can be decomposed into several parts:
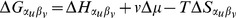
(3)The enthalpic contribution, 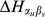, to the free energy of an oligomers (*i*-mer, where 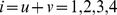) was determined as the minimum of the interaction energy of a given oligomer, which is schematically depicted with the enthalpic values in Figure 1. Naturally, the bigger the oligomer and the larger the patch, the stronger the interaction is. Importantly, due to the patch size, a tetramer of 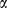-particles (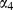) has no enthalpy gain compared to its trimer counterpart (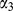).

The next contribution is the free energy associated with changes in the internal degrees of freedom of the protein molecule, 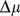; this value is large compared to 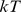 since the experimental measurements only report evidence for the 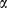 state in solution: no free molecules in the 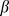 state are observed. The free energy difference is fixed at 15 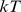 for each monomer which changes its state from the soluble 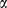 state to the 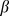 state that it assumes in the aggregates (for more details see methods).

The last contribution is entropic 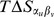, where 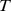 is the temperature and 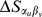 is the entropy; this contribution stems mainly from the loss of translational entropy upon binding to the cluster, but it also includes the rotational and internal entropy.

**Figure 7:**
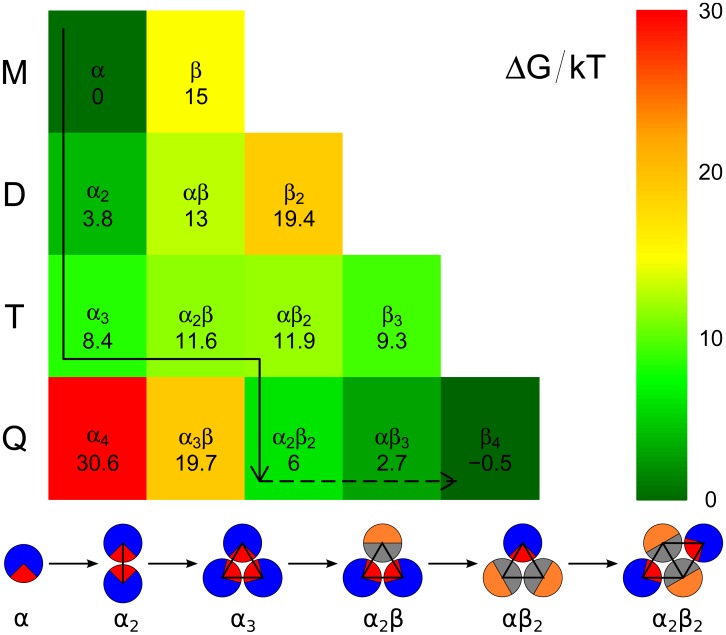
Free energy landscape for the formation of the oligomers with the marked free energy minimum path of the nucleus formation. The dashed line follows the free energy change to minimum within our largest cluster, where the cluster could also grow into a pentamer with an unknown free energy preference. The different oligomers are denoted as M: monomer, D: dimer, T: trimer, and Q: tetramer. The greek letters designate the composition of the clusters, where 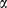 stand for random coil, while 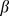 represents 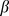-sheet. The free energy path is schematically depicted at the bottom of the figure, where the 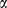 state is represented by a blue circle with a 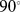 red patch and 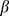 state is displayed as a half orange/half grey circle, which represents the interaction parameters of the employed model. The displayed values are for 0.19 mM concentration.

Two additional simulations were carried out, each with fixed type of achiral monomers (pure 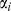 and pure 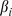). The free energy of the *i*-mers was determined based on their relative populations (see supplementary information Text S1). The enthalpic contributions can be determined directly by computing the relevant interaction terms in an ideal configuration. By subtracting it from the free energy and by dividing by 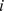 we obtain the entropy per monomer for each (pure) *i*-mer. Assuming that the entropy of a single particle in a given state is similar in all (mixed) *i*-mers independent of their composition, we can construct the free energy landscape of the oligomers 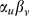; again using the enthalpic contribution for an ideal configuration (see Figure 7 and the supplementary information Text S1 for a detailed description of how to construct it). Note, that the achiral monomers were employed for this calculation as it easier to determine the ideal maximum interaction enthalpy and therefore enthalpic contribution to the free energy. The nucleation for achiral and chiral model is very similar as they differ mainly in the later stage as self-assembled fibrils in their rigidity.

The analysis of the energy landscape reveals the microscopic details through which primary nucleation occurs in the coarse grained system. The path of the lowest energy connecting the monomeric states with the aggregates starts with the assembly of a dimer 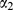 of molecules in an unstructured state. At this stage, the free energy cost for the conversion of one of the molecules in the dimer to the 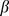 state is 9.2 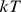, less than the cost of the conversion in solution, 15 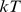, but still sufficiently high for this mechanism not to be the major contribution to the overall production of aggregates. However, the unstructured trimer 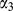, obtained through monomer addition from the dimer 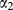, possesses a lower free energy than the mixed dimer 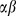, and at this stage the conversion of one of the molecules to the 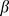 state is associated with a significantly lower cost of 3.2 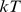 resulting in the species 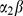. Subsequent conversions to the 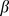 state are associated with an even smaller energy cost, and the species 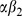 is only 0.3 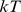 higher in free energy relative to the species 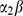. This mixed aggregate represents the species with the highest free energy on the aggregation pathway; subsequent additions of monomers to this nucleus lower its free energy and result in the formation of 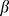-sheet fibrils. Therefore the nucleus size in the formulation of Eq. 1 is 4, in excellent agreement with the analysis performed on the average kinetic data. The first fully 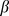-sheet aggregate is the tetramer 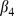.

The overall nucleation pathway that emerges from our simulations is that of a nucleation process followed by a conformational conversion. The conversion step is, however, dependent on the addition of monomers and does not take the form of one step cooperative conversion [36]. In particular the critical nucleus is a mixed, partially converted, aggregate rather than the fully unconverted species. This finding is likely to be of general value since the mechanism identified in the present study allows the conversion of a number of monomers in discrete steps combined with monomer addition steps to avoid the high free energy barrier associated with a conversion of the entire aggregate in a single step. These results therefore generalise the nucleated conformational conversion model [36] to include multi-step conversion.

## Discussion

We have devised a simulation scheme which allows us to study a system of aggregating peptides and compute quantities that are directly accessible in experiments, such as the scaling behaviour of the lag-time with aggregate concentration, and to relate these characteristics to the microscopic events that underlie the generation of single nuclei and their subsequent growth. In order to reach the long time scales required for such a study, we have employed a coarse-grained model, which includes a representation of the internal degrees of freedom of the polypeptide chain as well as the possibility to assemble into both oligomers and elongated fibrils. The obtained sigmoidal growth of the oligomers is in agreement with previous studies employing different two state models [25], [29].

The nucleus size was found to be in mutual agreement from all the employed methods. The most trivial one is the empirical observation of the oligomers' time distribution, where our results show that all tetramers develop into a fibril. The second method is the fit of the growth profile to Oosawa's theory, which has to be performed with data at varying initial monomer concentration for unambiguous results. The fitted nucleus size is 3.8. The same result was obtained from our analysis of the free energy landscape for the oligomer formation. The coarse grained system that we study possess parameters that are representative of experimental systems, where nucleus sizes of the order of 2–4 are commonly reported [6], [15], [51].

We found very good agreement of our simulated fibril growth with the theory of Oosawa, especially for the chiral model. The small deviations can be due to the fact that our simulation time is rather small (somewhere around 2 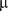
s, compared to hours in many experiments), a factor which required high concentrations in order to observe fibril growth. Some processes such as fibril fusion could, therefore, be enhanced under the conditions used in the simulations. Note that the current implementation of our model does not lead to any secondary nucleation [22], [23], [51] and the scale of our simulations prevents the fibril breakage [15], [52]. As a result we have not observed secondary nucleation pathways which would lead to different kinetics of fibril formation [15], [22].

The ability to compute the free energy landscape of the oligomers allowed us to study the sequence of events that leads to the generation of a fully 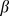-sheet aggregate from the monomeric 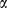 precursor state. We found that the that this conversion occurs concomitantly with the growth of the oligomers through monomer addition. Furthermore, the critical nucleus is found to be a mixed species including both converted and unconverted peptides, and its size is in good agreement with that determined from the analysis of the scaling behaviour of the average lag-time. This concerted mechanism allows the energy penalty from the conversion of individual peptides to be compensated by the energy gain from an increase in the number of favorable inter-peptide contacts.

## Supporting Information

Text S1 Supplementary information Text S1 contains detailed information about the calculation of free energy landscape and residual monomer concentration.
(ZIP)
