# Supplementary material for: Connecting Macroscopic Observables and Microscopic Assembly Events in Amyloid Formation Using Coarse Grained Simulations
Source: PLoS Comput Biol. 2012 Oct 11;8(10):e1002692. doi: 10.1371/journal.pcbi.1002692 (PMC3469425; doi:10.1371/journal.pcbi.1002692)
Supplement: Text S1 — Supplementary information Text S1 contains detailed information about the calculation of free energy landscape and residual monomer concentration. (ZIP) [file pcbi.1002692.s001.zip › TextS1.pdf]

Supplementary information

Noah S. Bieler[1], Tuomas P. J. Knowles[2], Daan Frenkel[2], Robert Vácha[2,3,*]
1 Laboratory for Physical Chemistry, ETH Zürich, Switzerland
2 Department of Chemistry, Lensfield Road, Cambridge, CB2 1EW, United Kingdom
3 National Centre for Biomolecular Research, Faculty of Science and CEITEC - Central European Institute of Technology, Masaryk University, Kamenice 5, 625 00 Brno-Bohunice, Czech Republic
∗ E-mail: Corresponding robertvacha@gmail.com

1 Free Energy Landscape

The free energy landscape was constructed using the following assumptions:

- The entropy of formation of an oligomer can be distributed equally into all its building units, since the entropy change compared to free monomer stems mainly from the loss of translational and rotational entropy (note, that β-particles in an oligomer can rotate more due to the larger patch size, without losing any enthalpy). This means the entropy change of an i-mer ($i = u + v$) is

$$\Delta S_{\alpha_u \beta_v} = u \Delta S_{1\alpha_{u+v}} + v \Delta S_{1\beta_{u+v}}, \tag{S1}$$

  where $\Delta S_{1\alpha_i}$ is the entropy change of one monomer in a pure α-i-mer.

- The oligomers are assumed to be in the minimum of interaction energy, where $\Delta H_{\alpha_u \beta_v}$ can be calculated by adding together all possible interactions (see Figure 6).

With the above assumptions the following procedure is used to construct the free energy landscape:

1. Calculate the free energy per monomer of pure α or β oligomers [1]:

$$\Delta G_{1i} = \frac{kT}{i} \ln \left(\frac{i x_1^i}{x_i} \right) \tag{S2}$$

2. Next the idealized enthalpic contribution is subtracted in order to obtain the entropic contribution to the oligomer ΔS_{α_i} and ΔS_{β_i} and per each monomer $\Delta S_{1\alpha_i} = \frac{\Delta S_{\alpha_i}}{i}$.

3. Using these terms an idealized entropic contribution for the oligomer is calculated using:

$$\Delta S_{\alpha_u \beta_v} = u \Delta S_{1\alpha_{u+v}} + v \Delta S_{1\beta_{u+v}} \tag{S3}$$

4. Finally the idealized enthalpic contribution $\Delta H_{\alpha_u \beta_v}$ as well as the chemical potential $\Delta\mu$ for the mixed oligomer is constructed from individual contributions:

$$\Delta G_{\alpha_u \beta_v} = \Delta H_{\alpha_u \beta_v} + v \Delta\mu - T \Delta S_{\alpha_u \beta_v} \tag{S4}$$

In order to test the the above assumptions further calculations were performed. In particular, we evaluated entropic contributions to dimers, trimers, and tetramers and compared them with our simulations.

The entropic contribution can be expressed in terms of the translational entropy of the monomer by following these steps: First we look at the free energy change for the aggregation of a pure dimer:

$$\Delta_r G_{12} = \Delta_r H_{12} - T \Delta_r S_{12} = \Delta H_2 - 2\Delta H_1 - T\Delta S_2 + 2T\Delta S_1 \tag{S5}$$

Here we apply the convention, that $\Delta_r Y_i$ means the change of Y in the reaction from an monomer to an i-mer and ΔY_{1i} is the contribution of one monomer in a i-mer to the value of Y, so that $\Delta_r Y_{1i} = i\Delta Y_{1i}$. Meanwhile ΔY_i is the contribution of i-mer to the value of Y.

Since $\Delta H_1 = 0$ and the entropy of the monomer is only constituted by the translational and rotational entropy, while the dimer has also some internal entropy, we can write:

$$\Delta_r G_{12} = \Delta H_2 - T\Delta S_2^{rot}(V) - T\Delta S_2^{tr}(V) - T\Delta S_2^{int}(V) + 2T\Delta S_1^{tr} + 2T\Delta S_1^{rot} \quad \text{(S6)}$$

If we assume, that $\Delta S_2^{tr}(V) \sim \Delta S_1^{tr}(V)$, we can rewrite the entropy loss $\Delta_r S_{12}$ as:

$$\Delta_r S_{12} = -\Delta S_1^{tr}(V) + \Delta S^{rest}, \quad \text{(S7)}$$

where we put the rest (the change in the rotational entropy and the internal entropy) into ΔS^{rest}. Applying the same steps for the free energy difference between three monomers and a trimer, we obtain:

$$\Delta_r S_{13} = -2\Delta S_1^{tr}(V) + \Delta S^{rest}. \quad \text{(S8)}$$

If we approximate $\Delta S^{rest} \approx 0$, we obtain $\Delta_r S_{13}(V) \approx 2\Delta_r S_{12}(V)$ or $\Delta S_{13} \approx \frac{4}{3}\Delta S_{12}$ for the entropy loss per monomer in a trimer and $\Delta S_{14} \approx \frac{3}{2}\Delta S_{12}$ for a monomer in a tetramer.

The only thing, which changes at different concentrations is the translational entropy, $\Delta\Delta S^{V_1,V_2}$, which can be calculated from the ideal gas approximation employing the equation of Sackur-Tetrode [2]:

$$S(V) = Nk \ln\left[\left(\frac{2\pi mkT}{h^2}\right)^{3/2} \frac{Ve^{5/2}}{N}\right] + S_{elec}, \quad \text{(S9)}$$

where N is the number of particles, m the mass, h Planck's constant and S_{elec} the electronic contribution to the entropy. Then, the entropy change between two different volumes is:

$$\Delta\Delta S_{theo}^{V_1,V_2} = \Delta S(V_2) - \Delta S(V_1) = \ln\left(\frac{V_2}{V_1}\right) = 3\ln\left(\frac{l_2}{l_1}\right) \quad \text{(S10)}$$

In the case, that the box length l_i is simply doubled, the entropy change is $\Delta\Delta S_{theo} = 3\ln(2) \approx 2.1$.

For the aggregation of monomer to a dimer or trimer we obtained follwing entropy changes per monomer:

$$\Delta\Delta S_{12}^{V_1,V_2} \approx -\frac{1}{2}\left(\Delta S_1^{tr}(V_2) - \Delta S_1^{tr}(V_1)\right) = -\frac{3}{2}\ln\left(\frac{l_2}{l_1}\right) \quad \text{(S11)}$$

$$\Delta\Delta S_{13}^{V_1,V_2} \approx -\frac{1}{3}\left(2\Delta S_1^{tr}(V_2) - 2\Delta S_1^{tr}(V_1)\right) = -2\ln\left(\frac{l_2}{l_1}\right) \quad \text{(S12)}$$

where subindex denotes the size of oligomer to which we are comparing (2 - dimer, 3 - trimer). In table S2 the above approximated values are compared to the simulations results and agreement is very good. Note: $\Delta\Delta S_{12}^{V_1,V_2} = -(1/2)\Delta\Delta S_{theo}$, $\Delta\Delta S_{13}^{V_1,V_2} = -(2/3)\Delta\Delta S_{theo}$ and $\Delta\Delta S_{14}^{V_1,V_2} = -(3/4)\Delta\Delta S_{theo}$.

Table S1. The free energy differences and the entropy loss between monomer and di-, tri- or tetrameric oligomer determined from simulations of fixed monemeric state (α and β). The values are per particle and units are in kT.

c/mM	$\Delta G_{12}^{(\alpha)}$	$\Delta G_{13}^{(\alpha)}$	$\Delta G_{14}^{(\alpha)}$	$\Delta G_{12}^{(\beta)}$	$\Delta G_{13}^{(\beta)}$	$\Delta G_{14}^{(\beta)}$
8.00	-0.30	-0.19	0.31	-1.26	-2.37	-2.91
2.37	0.53	0.99	1.60	-0.38	-1.13	-1.46
1.00	1.01	1.65	2.32	0.10	-0.47	-0.71
0.51	1.36	2.12	2.88	0.46	0.01	-0.16
0.30	1.65	2.50	3.24	0.74	0.39	0.26
0.19	1.88	2.81	3.45	0.98	0.70	0.62
c/mM	$T\Delta S_{12}^{(\alpha)}$	$T\Delta S_{13}^{(\alpha)}$	$T\Delta S_{14}^{(\alpha)}$	$T\Delta S_{12}^{(\beta)}$	$T\Delta S_{13}^{(\beta)}$	$T\Delta S_{14}^{(\beta)}$
8.00	-3.90	-8.21	-10.81	-2.94	-6.03	-7.59
2.37	-4.73	-9.39	-12.10	-3.82	-7.27	-9.04
1.00	-5.21	-10.05	-12.82	-4.30	-7.93	-9.79
0.51	-5.56	-10.52	-13.38	-4.66	-8.41	-10.34
0.30	-5.85	-10.90	-13.74	-4.94	-8.79	-10.76
0.19	-6.08	-11.21	-13.95	-5.18	-9.10	-11.12

Table S2. The differences between the entropy loss from one concentration to an other. In the very right column the theoretical values according to equation (S10) for $\Delta\Delta S_{theo} = 3\ln(l_2/l_1)$ are listed. The superscript (α) and (β) denote the state of the PSCs. Note: $\Delta\Delta S_{12}^{V_1,V_2} = -(1/2)\Delta\Delta S_{theo}$, $\Delta\Delta S_{13}^{V_1,V_2} = -(2/3)\Delta\Delta S_{theo}$ and $\Delta\Delta S_{14}^{V_1,V_2} = -(3/4)\Delta\Delta S_{theo}$.

c/mM	$\Delta\Delta S_{12}^{(\alpha)}$	$\Delta\Delta S_{13}^{(\alpha)}$	$\Delta\Delta S_{14}^{(\alpha)}$	$\Delta\Delta S_{12}^{(\beta)}$	$\Delta\Delta S_{13}^{(\beta)}$	$\Delta\Delta S_{14}^{(\beta)}$	$-\Delta\Delta S_{theo}$
8.00 - 2.37	-0.8	-1.2	-1.3	-0.9	-1.2	-1.4	-1.2
2.37 - 1.00	-0.5	-0.7	-0.7	-0.5	-0.7	-0.8	-0.9
1.00 - 0.51	-0.4	-0.5	-0.6	-0.4	-0.5	-0.5	-0.7
0.51 - 0.30	-0.3	-0.4	-0.4	-0.3	-0.4	-0.4	-0.5
0.30 - 0.19	-0.2	-0.3	-0.2	-0.2	-0.3	-0.4	-0.5

2 Residual Monomer Concentration

As can be seen in Figure 4. not all monomers are depleted from the bulk, though the growth function (Eq. 1) implies a total conversion of free monomers to fibrils. This originates from derivation, where the dissociation rate k_-, i.e. the rate of monomers dissociating from the fibrils, is approximated by zero.

To verify this assumption we have calculated the concentration of monomers at the end of our simulations, which is linked to the equilibrium constant k_-/k_+:

$$m(t_{eq})/m_p = c(t_{eq}) = \frac{k_-}{k_+}, \tag{S13}$$

where $m(t_{eq})$ is the monomer mass concentration, $c(t_{eq})$ is the equilibrium monomer concentration, and m_p is molar mass of monomer peptide. k_+ is the monomer association rate, i.e. rate constant for the binding of a monomer to a fibril. The monomer concentrations for simulations which reached equilibrium are listed in Table S3. All the values are in the same very low order of magnitude, which confirms the validity of the low depolymerization rate approximation and justifies the use of Eq. 1.

Table S3. The equilibrium monomer concentration for the simulations with different box sizes.

c_0/mM	Number of monomers	$c(t_{eq})/\mu M$
8.00	0.5	6.5
2.37	4.9	19.2
1.00	6.9	11.4
0.51	18.5	15.7

References

1. Israelachvili JN (2007) Intermolecular and Surface Forces. Academic Press, 2 edition.

2. McQuarrie D (2000) Statistical Mechanics. University Science Books, 2 edition.
